# The Modulatory Roles of *N*-glycans in T-Cell-Mediated Autoimmune Diseases

**DOI:** 10.3390/ijms19030780

**Published:** 2018-03-08

**Authors:** Ming-Wei Chien, Shin-Huei Fu, Chao-Yuan Hsu, Yu-Wen Liu, Huey-Kang Sytwu

**Affiliations:** 1 Department of Microbiology and Immunology, National Defense Medical Center, No.161, Section 6, Min Chuan East Road, Neihu, Taipei 114, Taiwan; chienmw0103@hotmail.com (M.-W.C.); winniefold@gmail.com (S.-H.F.); 2 Graduate Institute of Life Sciences, National Defense Medical Center, No. 161, Section 6, Min Chuan East Road, Neihu, Taipei 114, Taiwan; hsu.chaoyuan@gmail.com (C.-Y.H.); candy_77615@yahoo.com.tw (Y.-W.L.); 3 Molecular Cell Biology, Taiwan International Graduate Program, Academia Sinica, Taipei 115, Taiwan

**Keywords:** *N*-glycan, *O*-glycan, autoimmune disease, multiple sclerosis, inflammatory bowel disease systemic lupus erythematosus, type 1 diabetes mellitus

## Abstract

Glycosylation is a ubiquitous posttranslational modification of proteins that occurs in the endoplasmic reticulum/Golgi. *N*-glycans and mucin-type *O*-glycans are achieved via a series of glycohydrolase- and glycosyltransferase-mediated reactions. Glycosylation modulates immune responses by regulating thymocyte development and T helper cell differentiation. Autoimmune diseases result from an abnormal immune response by self-antigens and subsequently lead to the destruction of the target tissues. The modification of *N*-glycans has been studied in several animal models of T-cell-mediated autoimmune diseases. This review summarizes and highlights the modulatory effects of *N*-glycosylation in several autoimmune diseases, including multiple sclerosis, systemic lupus erythematosus, inflammatory bowel disease, and type 1 diabetes mellitus.

## 1. Introduction of Glycosylation

### 1.1. Biosynthesis of N- and O-Linked Glycosylated Molecules

Proteins of the eukaryotic cell surface and secretory proteins are posttranslationally modified with Asn (*N*)- and Ser/Thr (*O*)-linked glycans in the endoplasmic reticulum (ER) and Golgi apparatus via the action of a series of glycohydrolases and glycosyltransferases. Thus, the formation of complex glycans depends on the expression and activity of these ER/Golgi enzymes. The nutrient environment of the cells and metabolic supply of substrates also affect their diversity.

**Figure 1:**
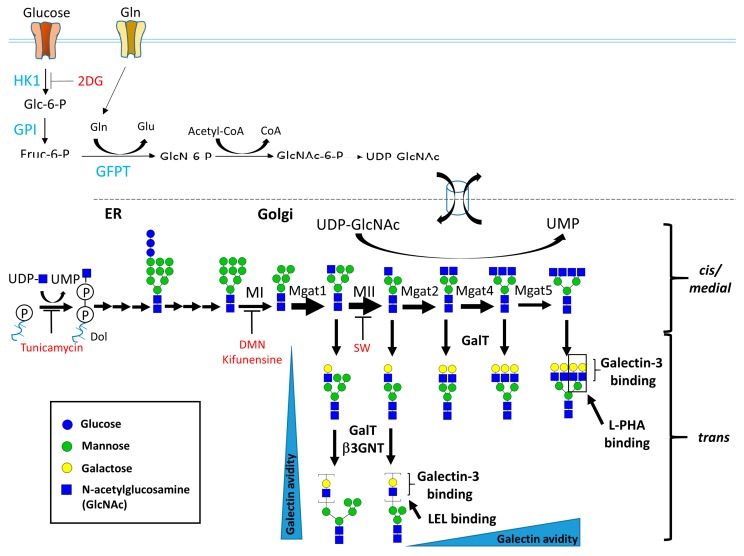
Biosynthesis of *N*-glycan branching. The UDP-GlcNAc supply is sensitive to glucose (Glc), glutamine (Gln), acetyl-CoA, and GlcNAc. UDP-GlcNAc is required for the initiation of *N*-glycans in the ER and is used for branching reactions in the Golgi. *N*-glycan branching is achieved via a series of glycohydrolase- and Mgat-mediated reactions. These *N*-glycans are further elongated with LacNAc units, which interact with galectin-3. These complex *N*-glycans are capped by sialic acid and fucose via the action of sialyltransferases and fucosyltransferases, respectively. 2DG = 2-deoxy-d-glucose, Dol = Dolichol, DMN = deoxymannojirimycin, MI = mannosidase I, MII = mannosidase II, SW = swainsonine. Additional structural modification via addition of sialic acid, fucose, *N*-acetylgalactosamine and/or sulfate is not shown.

*N*-Glycosylation occurs at N-X-S/T sites of proteins, which are sequentially modified via the action of α-mannosidases, *N*-acetyl-glucosaminyltransferases (Mgat) I, II, IV, and V (encoded by Mgat1, 2, 4, and 5, respectively). The Mgat family of proteins utilizes the same substrate, uridine diphosphate *N*-acetylglucosamine (UDP-GlcNAc), via the hexosamine biosynthesis pathway (HBP). Mgat1 transfers GlcNAc from UDP-GlcNAc to Man5GlcNAc2Asn of proteins to initiate the synthesis of complex and hybrid *N*-glycans. Mgat2 generates b1,2 GlcNAc branched *N*-glycans to form a bi-antennary structure. Mgat4a and Mgat5 further catalyze the β1,4 GlcNAc and b1,6 GlcNAc-branches of the mannose core of *N*-glycans to generate tri- and tetra-antennary structures, respectively (as illustrated in Figure 1). The Km values for UDP-GlcNAc decrease by ~400-fold between Mgat1 (0.04 mM) and Mgat5 (10 mM) [1,2]. In contrast, Mgat1 has a low affinity for the acceptor *N*-glycan at ~2 mM, and this relationship is reversed for Mgat4 and Mgat5. Both the deficiency of UDP-GlcNAc and Mgat1 overexpression have been shown to reduce *N*-glycan branching by inhibiting the actions of Mgat4 and Mgat5 [2,3]. Moreover, these Mgat functions depend on each other in the formation of complex *N*-glycans. The functionality of Mgat2, Mgat4a and Mgat5 requires Mgat1-mediated *N*-glycans. Mgat4a and Mgat5 cannot transfer GlcNAc to the β1,4 GlcNAc and b1,6 GlcNAc-branches of the mannose core of *N*-glycans only in the presence of Mgat2. The absence of Mgat5 will not influence other Mgat-mediated *N*-glycan processing.

*N*-glycan branching is further modified by β1,4 galactosyltransferases (β4Gal-Ts) and β1,3*N*-acetyl-glucosaminyltransferases (βGNTs), and elongated with poly *N*-acetyllactosamine (poly-LacNAc). Finally, the complex *N*-glycans are capped by sialic acid and fucose via the actions of sialyltransferases and fucosyltransferases, respectively (Figure 1). The complex *N*-glycans serve as ligands for a series of lectins, including galectins [4,5], siglecs [6], and selectins [7,8], that modulate the immune homeostasis.

**Figure 2:**
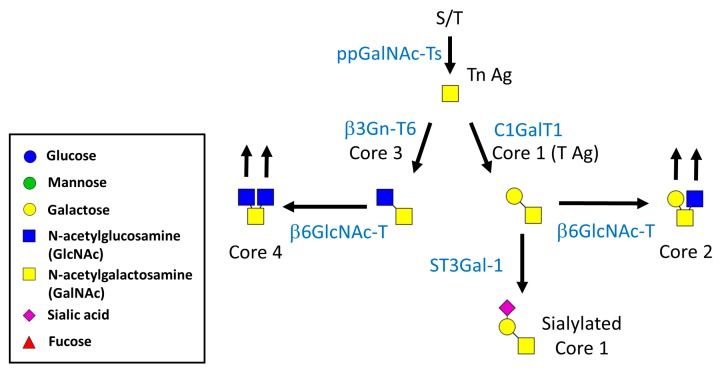
An overview of the mucin-type *O*-glycans. Mucin-type *O*-glycans are modified with a GalNAc sugar at the hydroxyl group of serine or threonine residues of proteins by ppGalNAc-Ts. The addition of galactose and GlcNAc results in the formation of the core 1 and core 3 structures, respectively. These core structures can be catalyzed by β6GlcNAc-Ts, which add GlcNAc in α1,6-linkage to the extant GalNAc (core 2 or core 4 structures). These glycans can be modified further via linear or branched structures through the addition of other sugars, such as galactose, GlcNAc, fucose, and sialic acid. Additional structural modification via addition of sialic acid, fucose, GlcNAc and Gal is not shown.

Mucin-type *O*-glycans are abundant on mucins and occur in the in proline, threonine, and serine repeating domains of proteins (i.e., PTS domains) [9,10,11]. These glycans are found on many cell-surface and -secreted proteins and play a critical role in recognition, adhesion, and communication when cells interact with lectins in the environment [9,12,13]. Mucin-type *O*-glycans are modified with a GalNAc sugar at the hydroxyl group of serine or threonine residues of proteins by a large family of polypeptide GalNAc transferases (ppGalNAc-Ts). The core 1 (T-antigen) structure is catalyzed by the core 1 α1,3 galactosyltransferase (T-synthase or C1GalT1), which adds a galactose in a β1,3-linkage to the extant GalNAc [14,15]. Cosmc, the ER chaperone, is responsible for the folding and activity of the mammalian C1GalT1 enzyme, and Cosmc deficiency selectively inhibits the synthesis of core 1 *O*-glycans in mammals, suggesting that Cosmc is essential for the activity of the C1GalT1 enzyme [16,17,18]. The core 3 structure is catalyzed by β1,3-*N*-acetylglucosaminyltransferase 6, which adds a GlcNAc in β1,3-linkage to the extant GalNAc of proteins. The core 1 and core 3 structures can be catalyzed further by β1,6-*N*-acetylglucosaminyltransferases, which add GlcNAc in α1,6-linkage to the extant GalNAc (core 2 or core 4 structures). These glycans can be further extended via linear or branched structures through the addition of other sugars, such as galactose, GlcNAc, fucose, and sialic acid (Figure 2).

Galectins, a family of LacNAc-binding animal lectins, contain at least one conserved carbohydrate-recognition domain (CRD) and are expressed ubiquitously in the extracellular matrix, at the cell surface and in the cytosol [5,19,20,21]. In general, they are classified as: prototype galectins (such as galectin 1), which contain one CRD and occur as monomers or dimers; tandem repeat-type galectins (such as galectin 9), which have two different CRDs connected by a linker; and the chimera-type (such as galectin-3), which contains C-terminal CRD conjugated to a non-lectin domain at the N-terminus [22]. Most galectins interact with *N*-glycans at the cell surface to form lattices [23,24,25] and enhance glycoprotein retention time at the cell surface [2,26]. Galectin-3 interacts preferentially with Mgat5-mediated *N*-glycans of TCR molecules on T cells [26]. Interestingly, loss of Mgat2 decreases *N*-glycan branching significantly at the cell surface compared with Mgat5 deletion, and the interaction of galectin-3 with the total LacNAc content at the cell surface in Mgat2^−/−^ and Mgat5^−/−^ T cells is similar. The compensatory effects of the LacNAc content in Mgat2^−/−^ T cells preferentially occur via the extension of poly-LacNAc in Mgat1-mediated *N*-branching [27]. This finding indicates that severe *N*-glycan branching deficiency results in linear extension with poly-LacNAc structures on *N*-glycans.

### 1.2. Biological Functions of N-Glycosylation

Mgat1 is responsible for generating the first antennary. *Mgat1*^−/−^ mouse embryos lacking all *N*-glycan branching of proteins exhibited developmental retardation and died between 9.5 and 10.5 days [28,29]. Mgat2 is responsible for generating the second antenna of *N*-glycans. Loss of Mgat2 in mice resulted in defective complex *N*-glycans and revealed a novel bisecting *N*-glycans structure [30]. Mice lacking Mgat2 displayed severe gastrointestinal, hematologic, and osteogenic defects, which are comparable to human Mgat2 deficiency (CDG-II) [31]. Mgat4a is required for the formation of the tri-antennary *N*-glycans of proteins, and these glycans interact with galectin-9 in pancreatic β cells. Mice lacking Mgat4a displayed hyperglycemia, obesity, and insulin resistance in response to a high-fat diet [32]. In β-cell-specific *Mgat4a* transgenic mice, β cells increased insulin sensitivity and protected against type 2 diabetes [33]. These findings suggest that Mgat4a-mediated *N*-glycan branching on Glut2 preferentially binds to galectin-9 and enhances surface retention. Mgat4a and Mgat5 are responsible for the formation of tri- and tetra-antenna *N*-glycans structures, respectively. Interestingly, Mgat5-deficient mice were slightly hypoglycemic and were hypersensitive to fasting [34,35]. The insulin responses were normal in Mgat5-deficient mice, but the higher levels of glucagon contributed to their lean phenotype [35]. Moreover, Mgat5 was upregulated in carcinomas for cytokine signaling and subsequently affected the epithelial-mesenchymal transition (EMT), cell motility, and tumor metastasis [34]. Galectin-3 preferentially interacted with the poly-LacNAc structure on the Mgat5-mediated *N*-glycan branching on EGFR and TGF-βRII at the cell surface, and delayed endocytosis [36]. These studies identified the lattice as a key regulator of the receptors that can modify cell growth and the inflammatory response.

### 1.3. Effect of N-Glycans on the Immune System

Many studies have proven that *N*-glycan branching plays a major role in the immune system [26,30,37]. During thymocyte development, the levels of *N*-glycan branching vary by 5-fold from double negative (DN) to single positive (SP) thymocytes, followed by a decline in the levels of *N*-glycan branching of 2-fold from SP to peripheral T cells [38], suggesting that these changes in *N*-glycan branching regulate TCR clustering during thymocyte development. The number of T cells in the thymus and spleen of mice lacking *N*-glycan branching in T cells lacking Mgat1 [38] or Mgat2 [30,38] was significantly reduced after the increase in co-receptor endocytosis and the inhibition of Lck activation. Therefore, complex *N*-glycans are required for the interactions between peptide-MHC and TCR for positive selection during thymocyte development. Mgat5 is responsible for the formation of tetra-antennary *N*-glycan, and its deficiency did not significantly alter the number and population of T cells in the thymus and spleen. Mgat5 deficiency decreased the lattice formed between T cells and galectin-3 and increased TCR clustering in immune synapses; subsequently, it contributed to the development of delay-type hypersensitivity (DTH) and experimental autoimmune encephalomyelitis (EAE) [26]. Mgat5-mediated *N*-glycans further attenuated Th1 [39] and Th17 cell differentiation [40] and increased the surface retention of cytotoxic T lymphocyte antigen 4 (CTLA-4) [2]. CTLA-4, which is an inhibitory costimulatory molecule that plays a major role in T-cell arrest. The Thr17Ala polymorphism in human CTLA-4, which reduces the number of its *N*-glycan sites from two to one, is associated with multiple sclerosis (MS) risk via the attenuation of its surface retention [3,41,42]. Cytokines and their receptor-associated activation of signal transducer and activator of transcription proteins (STATs) regulate the expression of specific transcriptional factors and contribute to T helper cell development [43,44]. IL-2Rα (CD25), which is expressed mainly on activated T cells or Treg cells, plays a critical role in the high-affinity binding to IL-2, in conjunction with IL-2Rβ and the γ chain. This binding activates MAPK, PI3K/Akt/mTOR, and STAT5 to regulate T-cell survival, proliferation, differentiation, and activation-induced cell death (AICD). CD25 is a highly glycosylated protein that contains *N*- and mucin-type *O*-glycan modifications [43,45]. Inhibition of *N*-glycosylation by glucosamine or tunicamycin treatment significantly attenuates the surface retention of CD25 on T cells and IL-2 downstream signaling. These inhibitory effects of CD25 systemically downregulate Th1, Th2, and Treg cell differentiation and markedly promote Th17 cell development [45,46]. Moreover, supplementation with glucose and glutamine increases *N*-glycosylation biosynthesis and subsequently enhances the surface retention of CD25 through the HBP [46]. Interestingly, the inhibition of *N*-glycosylation moderately attenuates the surface retention of other cytokine receptors including IL-12Rβ2, IL-4Rα and IL-6R and slightly downregulates the activation of STAT4, STAT6, and STAT3, respectively.

Why is the surface retention of CD25 on T cells significantly affected by the inhibition of *N*-glycans or the nutrients in the HBP? The number of *N*-glycan modifications on each glycoprotein sequence regulates the surface retention of glycoproteins [2,36]. The predicted molecular weight of CD25 is 25 kDa, and its real molecular weight is approximately 55 kDa. Nonetheless, CD25 contains three consensus *N*-glycosylation sites, and the response of CD25 was similar to those that were decorated with a high number of *N*-glycans [36]. Another possibility is elevated expression of CD25 on T cells compared with the expression of other cytokine receptors, such as IL-12Rβ2 and IL-4Rα. These findings suggest that both the number of *N*-glycans and the expression levels of receptors contribute to T cell growth and differentiation.

## 2. Overview of Autoimmune Diseases and the Modulatory Effects of *N*-Glycan Branching on These Diseases

Autoimmune diseases, which result from immune-system disorders caused by self-antigens, affect approximately 3–8% of the population. Over 80 autoimmune disorders have been identified, and genetic and environmental factors have been shown to contribute to the development of autoimmunity in humans and animal studies. The activation of immune cells, such as T or B cells, by self-antigens in tissues leads to the secretion of inflammatory cytokines or autoantibodies, which damage the target cells or tissues [47]. In this section of the article, we will review the modulatory effects of *N*-glycan branching in several autoimmune diseases, including MS, systemic lupus erythematosus (SLE), inflammatory bowel disease (IBD), and type 1 diabetes (T1D).

### 2.1. Multiple Sclerosis

#### 2.1.1. Pathogenesis and Experimental Animal Models of MS

Multiple sclerosis (MS) is a neurodegenerative disease of the central nervous system (CNS) [48]. Immune cells, such as macrophages, neutrophils and T cells, are involved in the damage of the neuronal myelin sheath that occurs during the development of MS [49,50]. Experimental autoimmune encephalomyelitis (EAE) is the mouse model of MS because of its pathological similarity to human multiple sclerosis [51]. EAE can be induced in susceptible strains of mice, such as SJL and C57BL/6 mice, by immunization with the proteolipid protein (PLP) or myelin oligodendrocyte glycoprotein (MOG) emulsified in complete Freund’s adjuvant, respectively [52]. CD4 T cells play a critical role in the development of MS. Inflammatory cytokines, such as IFN-γ [53,54] and IL-17 [55], are secreted by these cells and contribute to the severe inflammation and damage of the myelin sheath and neurons observed in MS.

#### 2.1.2. Role of *N*-Glycan Branching on T Cells in MS

Mgat5 catalyzes the addition of β1,6GlcNAc to *N*-glycan, to form a tetra-antennary structure. Mgat5-deficient mice display enhanced DTH and increase susceptibility to EAE via the downregulation of the threshold to TCR clustering [26], the enhancement of IFN-γ production by T cells [39], and the decrease in CTLA-4 surface retention [2]. T-cell-specific Mgat2-deficient mice (Mgat2^f/f^/Lck-Cre) also display a significantly more severe EAE than do their control littermates [27]. Interestingly, Mgat2 deficiency leads to a marked decrease in *N*-glycan branching at the cell surface compared with Mgat5 deficiency, whereas the interaction of galectin-3 with the total LacNAc content at cell surface is similar in Mgat2^−/−^ and Mgat5^−/−^ T cells. The compensatory effects of the LacNAc content in Mgat2^−/−^ T cells preferentially occurs via the extension of poly-LacNAc of Mgat1-mediated *N*-glycan branching. Mgat2^f/f^/Lck-Cre mice were further treated with kifunensine, which inhibits α-mannosidase I and blocks the poly-LacNAc context of Mgat1-mediated *N*-glycan branching, resulting in a dramatic increase in clinical score. These findings demonstrate that poly-LacNAc extension plays a major role in the control of T cell growth, differentiation, and autoimmunity.

Several mouse inbred strains, such as PL/J and SJL mice, have an intrinsic deficiency in *N*-glycan branching in T cells compared with other inbred strains, such as BALB/c and C57BL/6 mice. PL/J mice display the lowest levels of *N*-glycan branching vs. other inbred mouse strains [56]. Mgat5-deficient PL/J mice spontaneously develop inflammatory demyelination and neurodegeneration and display Tim-3^+^ Th1 cells more frequency than do PL/J mice, which is consistent with the regulation of Th1 cell differentiation by *N*-glycan branching in vitro [39]. Several polymorphisms of Mgat1 that increase the levels of mRNA and enzymatic activity reduce the UDP-GlcNAc utilization by Mgat4 and Mgat5, and result in a decrease in *N*-glycan branching [3]. CTLA-4 is an inhibitory molecule that has a higher affinity for CD80/CD86 on antigen-presenting cells and negatively regulates the T-cell response [57,58,59]. CTLA-4 has two *N*-glycosylation sites. The Thr17Ala polymorphism in human CTLA-4, which results in one *N*-glycosylation site, not only limits the surface retention of CTLA-4 on T cells but also represents a risk factor of MS [3]. Both GlcNAc supplementation and vitamin D treatment increase *N*-glycan branching and enhance the surface retention of CTLA-4 on T cells, subsequently attenuating the development of demyelinating disease. Thus, the genetic defect of *N*-glycan branching in mouse or human T cells is directly associated with MS, and metabolic supplementation may increase *N*-glycan branching on T cells and reduce the risk of MS.

Glucose, glutamine and GlcNAc are metabolites for the biosynthesis of UDP-GlcNAc in the HBP. UDP-GlcNAc is further used in the ER for the initiation of *N*-glycosylation and in the Golgi for the generating *N*-glycan branching. Glucosamine, an amino sugar, is also the substrate of UDP-GlcNAc in HBP [60,61] and interferes with the process of *N*-linked glycosylation [62,63], unlike GlcNAc. Many studies have shown that glucosamine has immunomodulatory effects on autoimmune diseases [64,65,66,67]. We first demonstrated that glucosamine treatment inhibits Th1, Th2, and Treg cell differentiation and markedly promotes Th17 polarization by interfering with the *N*-glycosylation of CD25 and downregulating its downstream signaling. These effects of glucosamine were similar to those observed in tunicamycin-incubated cells. Interestingly, excess glucose rescues this glucosamine-mediated regulation, suggesting a functional competition between glucose and glucosamine. Moreover, low-dosage of glucosamine treatment exacerbates the severity of EAE by enhancing Th17 cell differentiation [45], which is consistent with previous studies demonstrating an inhibitory effect of *N*-glycan branching on EAE development. Furthermore, another study has shown that glucose and glutamine treatments not only block Th17 cell differentiation but also induce a cell-fate switch to iTreg cells via the increase of *N*-glycan branching and the surface retention of CD25 [46]. These findings further provide evidence that glycolysis and glutaminolysis cooperatively modulate T-cell development, differentiation, and self-tolerance through limited supply to *N*-glycan biosynthesis.

### 2.2. Systemic Lupus Erythematosus

#### 2.2.1. Pathogenesis and Experimental Animal Models of Systemic Lupus Erythematosus

Systemic lupus erythematosus (SLE) is a complex autoimmune disease that results from the interaction between the innate and the adaptive immunity. This disease syndrome includes malar (“butterfly”), rash, photosensitivity, nephritis and arthritis caused by the overproduction of autoantibodies to nuclear antigens and the formation of immune complexes [68]. Genetic, environmental, hormonal, epigenetic, and immunoregulatory factors are involved in the damage of multiple tissues observed during the development of SLE. Inflammatory cytokines, such as IL-2, IL-6, IFN-γ, IL-17 and TNF-α, are involved in the pathogenesis of the disease [69]. Although SLE was considered to be a Th1-mediated disorder [70], IL-6 and IL-17 also contribute to the disease [71], suggesting that the Th1 and Th17 responses play critical roles in the development of SLE.

The various mouse models of SLE include the NZB/W F1 hybrids between the New Zealand Black (NZB) and New Zealand White (NZW) strains, BXSB/*Yaa* strains, and MRL-Fas^lpr^ strains.

The NZB/W F1 strains are the classical model of SLE. Both NZB and NZW display a normal phenotype, whereas NZB/W F1 strains develop severe SLE-like phenotypes, which include lymphadenopathy, splenomegaly, antinuclear autoantibodies, and immune complex-mediated glomerulonephritis (GN) [72]. GN develops apparently at 5–6 months of age and results in kidney failure and death at 10–12 months of age. Unlike human SLE and the MRL/*lpr* mouse models, the autoantibodies from NZB/W F1 mice do not act against RNA-containing complexes.

MRL-Fas^lpr^ mice are another mouse model of SLE-like autoimmune syndromes. The *lpr* mutation alters the transcription of Fas, which is a surface-bound receptor that interacts with the Fas ligand. The deficiency of Fas signaling on B cells and T cells from MRL-Fas^lpr^ mice results in a defect in apoptosis. These mice spontaneously display systemic autoimmunity, massive lymphadenopathy, arthritis, and immune complexes [73,74].

#### 2.2.2. Role of *N*-Glycan Branching in SLE

α-mannosidase II is a key enzyme that removes mannose residues from hybrid *N*-glycans, subsequently allowing Mgat2, Mgat4, and Mgat5 to generate complex *N*-glycans. α-mannosidase II-deficient mice display a systemic autoimmune disorder that is similar to human SLE [75]. The formation of defective *N*-glycans caused by α-mannosidase II deficiency leads to the generation of mannose-dependent glycan ligands for multiple innate lectin receptors [37]. Interestingly, Mgat5-deficient mice have defective of tetra-antennary *N*-glycans and display a spontaneous increase in leukocyte infiltrates in kidney at one year of age. These mice have mononuclear infiltrates and accumulation of fibrin, leading to the obliteration of Bowman’s space, which is indicative of autoimmune-mediated GN [26]. These findings suggest that defective *N*-glycan branching disrupts the defense mechanism of vertebrates that is involved in the distinction between the glycomes of lower eukaryotic and prokaryotic pathogens.

### 2.3. Inflammatory Bowel Disease

#### 2.3.1. The Pathogenesis and Animal Models for Inflammatory Bowel Disease

Inflammatory bowel disease (IBD) is a chronic immune-mediated disorder of the gastrointestinal tract that is mediated by genetic, immune, and environment factors and microbiota. Both Crohn’s disease (CD) and ulcerative colitis (UC) are IBDs; however, they exhibit distinct clinical features [76,77]. For example, Th1/Th17 cells and related cytokines, such as IFN-γ and IL-23, play critical roles in the pathogenesis of CD, whereas Th2-related cytokines, such as IL-5 and IL-13, contribute to the development of UC [78,79].

The various animal models of IBD include chemically induced mouse models, cell-transfer models, and genetically modified mice. The chemically induced mouse models include trinitrobenzene sulfonic acid (TNBS)-induced colitis, dextran sulfate sodium (DSS)-induced colitis, and oxazolone colitis. The cell-transfer model is initiated by adoptive transfers of (naïve) CD4^+^CD45RB^high^ T cells into T- and B-cell-deficient mice. The genetically engineered models include IL-10-deficiency colitis, T-bet transgenic mice, and T-cell specific Blimp-1 deficiency colitis [76]. These disease models have not only provided novel concepts regarding the pathogenesis of IBD but have also led to the development of potential therapeutic strategies.

#### 2.3.2. Role of Glycosylation on T Cells in IBD

Galectin-4 binds to memory CD4 T cells under conditions of intestinal inflammatory disorder in patients with UC via an interaction with the immature core-1 *O*-glycan at cell surface. This colitis-associated expansion effect of *O*-glycan on CD4 T cell expansion results in the clinical severity observed in T cell-mediated intestinal inflammation in mouse models because of the increase of immunological synapses [80].

In addition to the role of core-1 *O*-glycan in colitis, patients with UC exhibit defective *N*-glycan branching on TCR in lamina propria T cells [81]. This deficiency in *N*-glycan branching is caused by reduced *MGAT5* gene expression, which is associated with T-cell hyperactivation [26]. Fucose, which is a hydrophobic monosaccharide, can be modified with glycans based on different linkages including α1–2/1–3/1–4 (Lewis type), α1–6 (core fucosylation), and *O*-fucosylation. α1–6 fucosyltransferase (Fut8) plays a major role in the core fucosylation of *N*-glycans in proteins. The levels of FUT8-mediated core fucosylation on T cells are increased in inflamed intestinal mucosa of colitic mice and patients with IBD. Furthermore, Fut8-deficient mice are resistant to the development of colitis in chemical and cell-transfer induced mouse models, via the inhibition of TCR signaling [82]. These findings indicate that not only core-1 *O*-glycan, but also the branching and core fucosylation levels of *N*-glycans on T cells, are involved in the pathogenesis of IBD.

### 2.4. Type 1 Diabetes Mellitus

#### 2.4.1. Pathogenesis of Type 1 Diabetes Mellitus and Non-Obese Diabetic Mice

Type 1 diabetes (T1D) is an autoimmune diabetes that results from the T-cell-mediated destruction of insulin-producing β cells in pancreatic islets [83]. T1D is usually diagnosed in childhood and is also called juvenile diabetes. This disease is characterized by hyperglycemia, ketosis, insulitis, and the presence of anti-islet autoantibodies. Genetic and environmental factors contribute to the susceptibility and pathogenesis of T1D [84,85].

Non-obese diabetic (NOD) mice spontaneously develop autoimmune diabetes because of its pathological similarity to T1D [86]. Eighty to 90 % of female and 40–50% male NOD mice develop diabetes by the age of 30 weeks [84], while human T1D occurs at a frequency that is approximately equal between men and women. Treatment of female NOD mice with androgens significantly protects against insulitis and delays the development of diabetes [87], indicating a modulatory effect response on the endocrine on immune system.

Many studies have demonstrated that Th1 cells play a critical role in the pathogenesis of autoimmune diabetes. Treatment with neutralizing antibodies against IFN-γ [88] or IFN-γR-deficient [89] NOD mice delays the onset and prevents the incidence of diabetes. In addition, treatment of NOD mice with Th2 cytokines, IL-4, or IL-10 prevents insulitis and the onset of diabetes [90,91].

#### 2.4.2. Role of *N*-Glycans in T1D

PL/J and SJL mice display intrinsic deficiency in *N*-glycan branching on T cells compared with BALB/c and C57BL/6 mice [56]. Oral GlcNAc supplementation in NOD mice increases the *N*-glycan branching on T cells and subsequently protects against the incidence of autoimmune diabetes [92]. Moreover, the *MGAT1* and *CTLA-4* variants inhibit *N*-glycan branching and decrease the surface retention of CTLA-4, respectively; these two gene variants are also found in T1D [93] and MS [3].

Interestingly, we proved that high dosage of glucosamine treatment significantly inhibits Th1 cell differentiation via the downregulation of the *N*-glycosylation of CD25 and Glut1. Glucosamine treatment further delays the onset of diabetes after BDC2.5 T-cell transfer, which expresses a transgenic TCR with specificity for the islet antigen chromogranin A and prolongs the survival of islet grafts in NOD recipients [45]. However, T cells from NOD mice exhibit ~30% less *N*-glycan branching than do Balb/c and B10.S mice [56]. This difference may contribute to the Th1 cell differentiation and the development of autoimmune diabetes observed in NOD mice. However, the inhibitory effect of glucosamine severely affects *N*-glycosylation biosynthesis, which significantly inhibits CD25 surface retention and downregulates its downstream signaling. Taken together, these findings suggest that *N*-glycosylation plays a dual role in the differentiation of T cells and the development of autoimmune diabetes.

## 3. Conclusions

**Figure 3:**
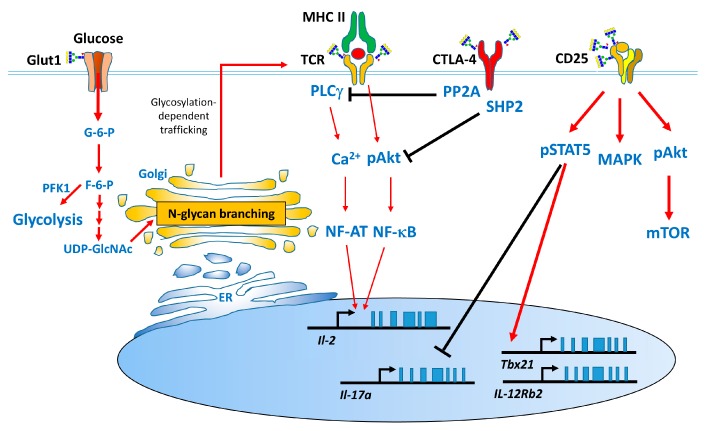
Metabolism, glycoprotein synthesis and T cell functions. Glucose is essential for *N*-glycosylation via its utilization to produce UDP-GlcNAc in the HBP. UDP-GlcNAc is used in the ER for the initiation of *N*-glycosylation and in the Golgi for generating *N*-glycan branching. TCR, CTLA-4, and CD25 are *N*-glycosylated. The *N*-glycan branching downregulates the threshold to TCR clustering and attenuates T cell activation. Moreover, the increase in CTLA-4 or CD25 surface retention present at the cell surface promotes downstream signaling and affects T cell functions. GLUT1, glucose transporter1; mTOR, mammalian target of rapamycin; PLCγ, Phospholipase Cγ; PP2A, Protein phosphatase 2; SHP-2, the SH2 domain-containing phosphatases.

This review summarizes findings that have led to a better understanding of the modulatory role of *N*-glycosylation on T cells (Figure 3) in several animal models and in patients with autoimmune diseases (Table 1). *N*-glycans play a critical role in T-cell development in the thymus and peripheral lymphoid organs. *N*-glycan branching on T cells not only prevents TCR clustering but also increases glycoprotein surface retention via interactions with galectins.

Patients with autoimmune diseases and many inbred mouse strains such as PL/J and NOD mice display intrinsic deficiency in *N*-glycan branching on T cells, and these defective *N*-glycans are associated with the development of autoimmune diseases. Oral supplementation with GlcNAc or vitamin D attenuates the development of EAE via an increase in *N*-glycan branching. Thus, the genetic defect of *N*-glycan branching in T-cell-associated autoimmune diseases may be restored by metabolite supplementation.

Because a series of enzymes in the ER/Golgi orchestrate the cascades of *N*-glycosylation synthesis in T lymphocytes, studying *N*-glycosylation and identifying its associated immune disorders has revealed important aspects of this regulatory machinery and may help provide new insights into the regulation of immune homeostasis and the potential of therapeutic strategies.

## Figures and Tables

**Table 1:** The modulatory effects of glycosylation in T-cell-mediated autoimmune diseases.

Disease	Strategies Used	Target Glycans	Clinical Outcome	Mechanisms	Ref.
EAE	T-cell specific Mgat2 KO mice	Defective *N*-glycan branching	Increase severity	Increased TCR clustering and CTLA-4 endocytosis	[27]
		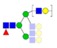			
	Mgat5 KO mice	Defective *N*-glycan branching	Increase severity	Increased TCR clustering and CTLA-4 endocytosis	[2,26]
		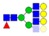			
	Administration of vitamin D	Enhance *N*-glycan branching	Reduced severity	Increased CTLA-4 surface retention	[3]
		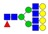			
	Administration of GlcNAc	Enhance *N*-glycan branching	Reduced severity	Decreased Th1 and Th17 cell responses	[40]
		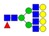			
	Administration of GlcN	Inhibition of *N*-glycosylation	Increase severity	Increased Th17 response via the decrease of CD25 surface retention	[45]
MS		*N*-glycan branching	Risk factor	Increased the TCR clustering and decreased CTLA-4 surface retention (Mgat1 haplotype)	[3]
		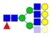			
		Decrease *N*-glycans site of CTLA-4	Risk factor	Increased CTLA-4 endocytosis (CTLA-4 SNP)	[3]
SLE	α-mannosidase II KO mice	*N*-glycan branching	Increase severity	Increased innate immunity	[37,75]
		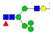			
	Mgat5 KO mice	*N*-glycan branching	Increase severity	Unknown	[26]
		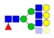			
IBD	Fut8 KO mice with DSS, TNBS and cell transfer-induced colitis	Defective Core fucosylation	Reduced severity	Decreased TCR signaling	[82]
		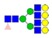			
	T-cell specific Tg C2GnT mice	Defective C2GnT	Reduced severity	Increased Immunological synapses	[80]
		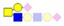			
T1D	Administration of GlcNAc	*N*-glycan branching	Reduced severity	Decreased Th1 responses	[92]
		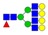			
	Administration of GlcN	Inhibition of *N*-glycosylation	Reduced severity	Decreased Th1 response via the downregulation of CD25 and Glut1 surface retention	[45]

C2GnT, core 2 β1,6-*N*-acetylglucosaminyltransferase; CTLA-4, cytotoxic T-lymphocyte-associated protein 4; DSS, dextran sodium sulfate ;EAE, experimental autoimmune encephalomyelitis; Fut, fucosyltransferase ;GlcN, glucosamine; GlcNAc, N-acetylglucosamine; IBD, inflammatory bowel disease; MS, multiple sclerosis; SLE, systemic lupus erythematosus; T1D, type 1 diabetes; TCR , T cell receptor; Th, T helper; TNBS, trinitrobenzene sulfonic acid.

## Abbreviations

EAE	Experimental autoimmune encephalomyelitis
HBP	Hexosamine biosynthesis pathway
SLE	Systemic lupus erythematosus
IBD	Inflammatory bowel disease
T1D	Type 1 diabetes mellitus
NOD	Non-obese diabetic
DSS	Dextran sodium sulfate
TNBS	Trinitrobenzene sulfonic acid

## References

[1] Sasai K., Ikeda Y., Fujii T., Tsuda T., Taniguchi N. (2002). UDP-GlcNAc concentration is an important factor in the biosynthesis of β1,6-branched oligosaccharides: Regulation based on the kinetic properties of *N*-acetylglucosaminyltransferase V. Glycobiology.

[2] Lau K.S., Partridge E.A., Grigorian A., Silvescu C.I., Reinhold V.N., Demetriou M., Dennis J.W. (2007). Complex *N*-glycan number and degree of branching cooperate to regulate cell proliferation and differentiation. Cell.

[3] Mkhikian H., Grigorian A., Li C.F., Chen H.L., Newton B., Zhou R.W., Beeton C., Torossian S., Tatarian G.G., Lee S.U. (2011). Genetics and the environment converge to dysregulate *N*-glycosylation in multiple sclerosis. Nat. Commun..

[4] Boscher C., Dennis J.W., Nabi I.R. (2011). Glycosylation, galectins and cellular signaling. Curr. Opin. Cell Biol..

[5] Cummings R.D., Liu F.T., Varki A., Cummings R.D., Esko J.D., Freeze H.H., Stanley P., Bertozzi C.R., Hart G.W., Etzler M.E. (2009). Galectins. Essentials of Glycobiology.

[6] Schnaar R.L. (2016). Glycobiology simplified: Diverse roles of glycan recognition in inflammation. J. Leukoc. Biol..

[7] Mitoma J., Bao X., Petryanik B., Schaerli P., Gauguet J.M., Yu S.Y., Kawashima H., Saito H., Ohtsubo K., Marth J.D. (2007). Critical functions of *N*-glycans in l-selectin-mediated lymphocyte homing and recruitment. Nat. Immunol..

[8] Rosen S.D. (2004). Ligands for l-selectin: Homing, inflammation, and beyond. Annu. Rev. Immunol..

[9] Bennett E.P., Mandel U., Clausen H., Gerken T.A., Fritz T.A., Tabak L.A. (2012). Control of mucin-type *O*-glycosylation: A classification of the polypeptide GalNAc-transferase gene family. Glycobiology.

[10] Steentoft C., Vakhrushev S.Y., Joshi H.J., Kong Y., Vester-Christensen M.B., Schjoldager K.T., Lavrsen K., Dabelsteen S., Pedersen N.B., Marcos-Silva L. (2013). Precision mapping of the human *O*-GalNAc glycoproteome through SimpleCell technology. EMBO J..

[11] Tran D.T., Ten Hagen K.G. (2013). Mucin-type *O*-glycosylation during development. J. Biol. Chem..

[12] Ellies L.G., Tsuboi S., Petryniak B., Lowe J.B., Fukuda M., Marth J.D. (1998). Core 2 oligosaccharide biosynthesis distinguishes between selectin ligands essential for leukocyte homing and inflammation. Immunity.

[13] Homeister J.W., Thall A.D., Petryniak B., Maly P., Rogers C.E., Smith P.L., Kelly R.J., Gersten K.M., Askari S.W., Cheng G. (2001). The α(1,3)fucosyltransferases FucT-IV and FucT-VII exert collaborative control over selectin-dependent leukocyte recruitment and lymphocyte homing. Immunity.

[14] Ju T., Brewer K., D’Souza A., Cummings R.D., Canfield W.M. (2002). Cloning and expression of human core 1 β1,3-galactosyltransferase. J. Biol. Chem..

[15] Ju T., Cummings R.D., Canfield W.M. (2002). Purification, characterization, and subunit structure of rat core 1 β1,3-galactosyltransferase. J. Biol. Chem..

[16] Ju T., Cummings R.D. (2002). A unique molecular chaperone Cosmc required for activity of the mammalian core 1 β3-galactosyltransferase. Proc. Natl. Acad. Sci. USA.

[17] Narimatsu Y., Ikehara Y., Iwasaki H., Nonomura C., Sato T., Nakanishi H., Narimatsu H. (2008). Immunocytochemical analysis for intracellular dynamics of C1GalT associated with molecular chaperone, Cosmc. Biochem. Biophys. Res. Commun..

[18] Wang Y., Ju T., Ding X., Xia B., Wang W., Xia L., He M., Cummings R.D. (2010). Cosmc is an essential chaperone for correct protein *O*-glycosylation. Proc. Natl. Acad. Sci. USA.

[19] Hsu D.K., Yang R.Y., Saegusa J., Liu F.T. (2015). Analysis of the intracellular role of galectins in cell growth and apoptosis. Methods Mol. Biol..

[20] Yang R.Y., Rabinovich G.A., Liu F.T. (2008). Galectins: Structure, function and therapeutic potential. Expert Rev. Mol. Med..

[21] Liu F.T., Rabinovich G.A. (2005). Galectins as modulators of tumour progression. Nat. Rev. Cancer.

[22] Dumic J., Dabelic S., Flogel M. (2006). Galectin-3: An open-ended story. Biochim. Biophys. Acta.

[23] Lee R.T., Lee Y.C. (2000). Affinity enhancement by multivalent lectin-carbohydrate interaction. Glycoconj. J..

[24] Brewer C.F., Miceli M.C., Baum L.G. (2002). Clusters, bundles, arrays and lattices: Novel mechanisms for lectin-saccharide-mediated cellular interactions. Curr. Opin. Struct. Biol..

[25] Ahmad N., Gabius H.J., Andre S., Kaltner H., Sabesan S., Roy R., Liu B., Macaluso F., Brewer C.F. (2004). Galectin-3 precipitates as a pentamer with synthetic multivalent carbohydrates and forms heterogeneous cross-linked complexes. J. Biol. Chem..

[26] Demetriou M., Granovsky M., Quaggin S., Dennis J.W. (2001). Negative regulation of T-cell activation and autoimmunity by Mgat5 *N*-glycosylation. Nature.

[27] Mkhikian H., Mortales C.L., Zhou R.W., Khachikyan K., Wu G., Haslam S.M., Kavarian P., Dell A., Demetriou M. (2016). Golgi self-correction generates bioequivalent glycans to preserve cellular homeostasis. Elife.

[28] Ioffe E., Stanley P. (1994). Mice lacking *N*-acetylglucosaminyltransferase I activity die at mid-gestation, revealing an essential role for complex or hybrid *N*-linked carbohydrates. Proc. Natl. Acad. Sci. USA.

[29] Metzler M., Gertz A., Sarkar M., Schachter H., Schrader J.W., Marth J.D. (1994). Complex asparagine-linked oligosaccharides are required for morphogenic events during post-implantation development. EMBO J..

[30] Wang Y., Tan J., Sutton-Smith M., Ditto D., Panico M., Campbell R.M., Varki N.M., Long J.M., Jaeken J., Levinson S.R. (2001). Modeling human congenital disorder of glycosylation type IIa in the mouse: Conservation of asparagine-linked glycan-dependent functions in mammalian physiology and insights into disease pathogenesis. Glycobiology.

[31] Tan J., Dunn J., Jaeken J., Schachter H. (1996). Mutations in the *MGAT2* gene controlling complex *N*-glycan synthesis cause carbohydrate-deficient glycoprotein syndrome type II, an autosomal recessive disease with defective brain development. Am. J. Hum. Genet..

[32] Ohtsubo K., Takamatsu S., Minowa M.T., Yoshida A., Takeuchi M., Marth J.D. (2005). Dietary and genetic control of glucose transporter 2 glycosylation promotes insulin secretion in suppressing diabetes. Cell.

[33] Ohtsubo K., Chen M.Z., Olefsky J.M., Marth J.D. (2011). Pathway to diabetes through attenuation of pancreatic β cell glycosylation and glucose transport. Nat. Med..

[34] Granovsky M., Fata J., Pawling J., Muller W.J., Khokha R., Dennis J.W. (2000). Suppression of tumor growth and metastasis in Mgat5-deficient mice. Nat. Med..

[35] Cheung P., Pawling J., Partridge E.A., Sukhu B., Grynpas M., Dennis J.W. (2007). Metabolic homeostasis and tissue renewal are dependent on β1,6GlcNAc-branched *N*-glycans. Glycobiology.

[36] Partridge E.A., Le Roy C., Di Guglielmo G.M., Pawling J., Cheung P., Granovsky M., Nabi I.R., Wrana J.L., Dennis J.W. (2004). Regulation of cytokine receptors by Golgi *N*-glycan processing and endocytosis. Science.

[37] Green R.S., Stone E.L., Tenno M., Lehtonen E., Farquhar M.G., Marth J.D. (2007). Mammalian *N*-glycan branching protects against innate immune self-recognition and inflammation in autoimmune disease pathogenesis. Immunity.

[38] Zhou R.W., Mkhikian H., Grigorian A., Hong A., Chen D., Arakelyan A., Demetriou M. (2014). *N*-glycosylation bidirectionally extends the boundaries of thymocyte positive selection by decoupling Lck from Ca^2+^ signaling. Nat. Immunol..

[39] Morgan R., Gao G., Pawling J., Dennis J.W., Demetriou M., Li B. (2004). *N*-acetylglucosaminyltransferase V (Mgat5)-mediated *N*-glycosylation negatively regulates Th1 cytokine production by T cells. J. Immunol..

[40] Grigorian A., Araujo L., Naidu N.N., Place D.J., Choudhury B., Demetriou M. (2011). *N*-acetylglucosamine inhibits T-helper 1 (Th1)/T-helper 17 (Th17) cell responses and treats experimental autoimmune encephalomyelitis. J. Biol. Chem..

[41] Anjos S., Nguyen A., Ounissi-Benkalha H., Tessier M.C., Polychronakos C. (2002). A common autoimmunity predisposing signal peptide variant of the cytotoxic T-lymphocyte antigen 4 results in inefficient glycosylation of the susceptibility allele. J. Biol. Chem..

[42] Maurer M., Loserth S., Kolb-Maurer A., Ponath A., Wiese S., Kruse N., Rieckmann P. (2002). A polymorphism in the human cytotoxic T-lymphocyte antigen 4 (*CTLA4*) gene (exon 1 +49) alters T-cell activation. Immunogenetics.

[43] Minami Y., Kono T., Miyazaki T., Taniguchi T. (1993). The IL-2 receptor complex: Its structure, function, and target genes. Annu. Rev. Immunol..

[44] O’Shea J.J., Paul W.E. (2010). Mechanisms underlying lineage commitment and plasticity of helper CD4+ T cells. Science.

[45] Chien M.W., Lin M.H., Huang S.H., Fu S.H., Hsu C.Y., Yen B.L., Chen J.T., Chang D.M., Sytwu H.K. (2015). Glucosamine Modulates T Cell Differentiation through Down-regulating *N*-Linked Glycosylation of CD25. J. Biol. Chem..

[46] Araujo L., Khim P., Mkhikian H., Mortales C.L., Demetriou M. (2017). Glycolysis and glutaminolysis cooperatively control T cell function by limiting metabolite supply to *N*-glycosylation. Elife.

[47] Davidson A., Diamond B. (2001). Autoimmune diseases. N. Engl. J. Med..

[48] Steinman L. (1996). Multiple sclerosis: A coordinated immunological attack against myelin in the central nervous system. Cell.

[49] Sobel R.A., Blanchette B.W., Bhan A.K., Colvin R.B. (1984). The immunopathology of experimental allergic encephalomyelitis. II. Endothelial cell Ia increases prior to inflammatory cell infiltration. J. Immunol..

[50] Rumble J.M., Huber A.K., Krishnamoorthy G., Srinivasan A., Giles D.A., Zhang X., Wang L., Segal B.M. (2015). Neutrophil-related factors as biomarkers in EAE and MS. J. Exp. Med..

[51] Ercolini A.M., Miller S.D. (2006). Mechanisms of immunopathology in murine models of central nervous system demyelinating disease. J. Immunol..

[52] Miller S.D., Karpus W.J. (2007). Experimental autoimmune encephalomyelitis in the mouse. Curr. Protoc. Immunol..

[53] Kennedy K.J., Karpus W.J. (1999). Role of chemokines in the regulation of Th1/Th2 and autoimmune encephalomyelitis. J. Clin. Immunol..

[54] Olsson T. (1992). Cytokines in neuroinflammatory disease: Role of myelin autoreactive T cell production of interferon-γ. J. Neuroimmunol..

[55] Bettelli E., Carrier Y., Gao W., Korn T., Strom T.B., Oukka M., Weiner H.L., Kuchroo V.K. (2006). Reciprocal developmental pathways for the generation of pathogenic effector TH17 and regulatory T cells. Nature.

[56] Lee S.U., Grigorian A., Pawling J., Chen I.J., Gao G., Mozaffar T., McKerlie C., Demetriou M. (2007). *N*-glycan processing deficiency promotes spontaneous inflammatory demyelination and neurodegeneration. J. Biol. Chem..

[57] Chen L., Ashe S., Brady W.A., Hellstrom I., Hellstrom K.E., Ledbetter J.A., McGowan P., Linsley P.S. (1992). Costimulation of antitumor immunity by the B7 counterreceptor for the T lymphocyte molecules CD28 and CTLA-4. Cell.

[58] Leach D.R., Krummel M.F., Allison J.P. (1996). Enhancement of antitumor immunity by CTLA-4 blockade. Science.

[59] Alegre M.L., Frauwirth K.A., Thompson C.B. (2001). T-cell regulation by CD28 and CTLA-4. Nat. Rev. Immunol..

[60] Jeon J.H., Suh H.N., Kim M.O., Ryu J.M., Han H.J. (2014). Glucosamine-induced OGT activation mediates glucose production through cleaved Notch1 and FoxO1, which coordinately contributed to the regulation of maintenance of self-renewal in mouse embryonic stem cells. Stem Cells Dev..

[61] Macauley M.S., Vocadlo D.J. (2010). Increasing *O*-GlcNAc levels: An overview of small-molecule inhibitors of *O*-GlcNAcase. Biochim. Biophys. Acta.

[62] Chen C.L., Liang C.M., Chen Y.H., Tai M.C., Lu D.W., Chen J.T. (2012). Glucosamine modulates TNF-α-induced ICAM-1 expression and function through *O*-linked and *N*-linked glycosylation in human retinal pigment epithelial cells. Investig. Ophthalmol. Vis. Sci..

[63] Klenk H.D., Scholtissek C., Rott R. (1972). Inhibition of glycoprotein biosynthesis of influenza virus by d-glucosamine and 2-deoxy-d-glucose. Virology.

[64] Forchhammer L., Thorn M., Met O., Gad M., Weidner M.S., Claesson M.H. (2003). Immunobiological effects of glucosamine in vitro. Scand. J. Immunol..

[65] Kim C.H., Cheong K.A., Park C.D., Lee A.Y. (2011). Glucosamine improved atopic dermatitis-like skin lesions in NC/Nga mice by inhibition of Th2 cell development. Scand. J. Immunol..

[66] Ma L., Rudert W.A., Harnaha J., Wright M., Machen J., Lakomy R., Qian S., Lu L., Robbins P.D., Trucco M. (2002). Immunosuppressive effects of glucosamine. J. Biol. Chem..

[67] Zhang G.X., Yu S., Gran B., Rostami A. (2005). Glucosamine abrogates the acute phase of experimental autoimmune encephalomyelitis by induction of Th2 response. J. Immunol..

[68] Tsokos G.C. (2011). Systemic lupus erythematosus. N. Engl. J. Med..

[69] Yap D.Y., Lai K.N. (2010). Cytokines and their roles in the pathogenesis of systemic lupus erythematosus: From basics to recent advances. J. Biomed. Biotechnol..

[70] Hayashi T. (2010). Therapeutic strategies for SLE involving cytokines: Mechanism-oriented therapies especially IFN-γ targeting gene therapy. J. Biomed. Biotechnol..

[71] Wong C.K., Lit L.C., Tam L.S., Li E.K., Wong P.T., Lam C.W. (2008). Hyperproduction of IL-23 and IL-17 in patients with systemic lupus erythematosus: Implications for Th17-mediated inflammation in auto-immunity. Clin. Immunol..

[72] Theofilopoulos A.N., Dixon F.J. (1985). Murine models of systemic lupus erythematosus. Adv. Immunol..

[73] Watson M.L., Rao J.K., Gilkeson G.S., Ruiz P., Eicher E.M., Pisetsky D.S., Matsuzawa A., Rochelle J.M., Seldin M.F. (1992). Genetic analysis of MRL-lpr mice: Relationship of the Fas apoptosis gene to disease manifestations and renal disease-modifying loci. J. Exp. Med..

[74] Perry D., Sang A., Yin Y., Zheng Y.Y., Morel L. (2011). Murine models of systemic lupus erythematosus. J. Biomed. Biotechnol..

[75] Chui D., Sellakumar G., Green R., Sutton-Smith M., McQuistan T., Marek K., Morris H., Dell A., Marth J. (2001). Genetic remodeling of protein glycosylation in vivo induces autoimmune disease. Proc. Natl. Acad. Sci. USA.

[76] Kaser A., Zeissig S., Blumberg R.S. (2010). Inflammatory bowel disease. Annu. Rev. Immunol..

[77] Xavier R.J., Podolsky D.K. (2007). Unravelling the pathogenesis of inflammatory bowel disease. Nature.

[78] Mizoguchi A., Mizoguchi E. (2010). Animal models of IBD: Linkage to human disease. Curr. Opin. Pharmacol..

[79] Duerr R.H., Taylor K.D., Brant S.R., Rioux J.D., Silverberg M.S., Daly M.J., Steinhart A.H., Abraham C., Regueiro M., Griffiths A. (2006). A genome-wide association study identifies IL23R as an inflammatory bowel disease gene. Science.

[80] Nishida A., Nagahama K., Imaeda H., Ogawa A., Lau C.W., Kobayashi T., Hisamatsu T., Preffer F.I., Mizoguchi E., Ikeuchi H. (2012). Inducible colitis-associated glycome capable of stimulating the proliferation of memory CD4+ T cells. J. Exp. Med..

[81] Dias A.M., Dourado J., Lago P., Cabral J., Marcos-Pinto R., Salgueiro P., Almeida C.R., Carvalho S., Fonseca S., Lima M. (2014). Dysregulation of T cell receptor *N*-glycosylation: A molecular mechanism involved in ulcerative colitis. Hum. Mol. Genet..

[82] Fujii H., Shinzaki S., Iijima H., Wakamatsu K., Iwamoto C., Sobajima T., Kuwahara R., Hiyama S., Hayashi Y., Takamatsu S. (2016). Core Fucosylation on T Cells, Required for Activation of T-Cell Receptor Signaling and Induction of Colitis in Mice, Is Increased in Patients With Inflammatory Bowel Disease. Gastroenterology.

[83] Kawasaki E., Abiru N., Eguchi K. (2004). Prevention of type 1 diabetes: From the view point of β cell damage. Diabetes Res. Clin. Pract..

[84] Wicker L.S., Todd J.A., Peterson L.B. (1995). Genetic control of autoimmune diabetes in the NOD mouse. Annu. Rev. Immunol..

[85] Sun J., Furio L., Mecheri R., van der Does A.M., Lundeberg E., Saveanu L., Chen Y., van Endert P., Agerberth B., Diana J. (2015). Pancreatic β-Cells Limit Autoimmune Diabetes via an Immunoregulatory Antimicrobial Peptide Expressed under the Influence of the Gut Microbiota. Immunity.

[86] Aoki C.A., Borchers A.T., Ridgway W.M., Keen C.L., Ansari A.A., Gershwin M.E. (2005). NOD mice and autoimmunity. Autoimmun. Rev..

[87] Fox H.S. (1992). Androgen treatment prevents diabetes in nonobese diabetic mice. J. Exp. Med..

[88] Debray-Sachs M., Carnaud C., Boitard C., Cohen H., Gresser I., Bedossa P., Bach J.F. (1991). Prevention of diabetes in NOD mice treated with antibody to murine IFN γ. J. Autoimmun..

[89] Yi Z., Li L., Garland A., He Q., Wang H., Katz J.D., Tisch R., Wang B. (2012). IFN-γ receptor deficiency prevents diabetes induction by diabetogenic CD4+, but not CD8+, T cells. Eur. J. Immunol..

[90] Cameron M.J., Arreaza G.A., Zucker P., Chensue S.W., Strieter R.M., Chakrabarti S., Delovitch T.L. (1997). IL-4 prevents insulitis and insulin-dependent diabetes mellitus in nonobese diabetic mice by potentiation of regulatory T helper-2 cell function. J. Immunol..

[91] Pennline K.J., Roque-Gaffney E., Monahan M. (1994). Recombinant human IL-10 prevents the onset of diabetes in the nonobese diabetic mouse. Clin. Immunol. Immunopathol..

[92] Grigorian A., Lee S.U., Tian W., Chen I.J., Gao G., Mendelsohn R., Dennis J.W., Demetriou M. (2007). Control of T Cell-mediated autoimmunity by metabolite flux to *N*-glycan biosynthesis. J. Biol. Chem..

[93] Yu Z., Li C.F., Mkhikian H., Zhou R.W., Newton B.L., Demetriou M. (2014). Family studies of type 1 diabetes reveal additive and epistatic effects between MGAT1 and three other polymorphisms. Genes Immun..

